# Evaluation of Beta-Defensin 1 and Mannose-Binding Lectin 2 Polymorphisms in Children with Dental Caries Compared to Caries-Free Controls: A Systematic Review and Meta-Analysis

**DOI:** 10.3390/children10020232

**Published:** 2023-01-28

**Authors:** Ghazal Hemati, Mohammad Moslem Imani, Parsia Choubsaz, Francesco Inchingolo, Roohollah Sharifi, Masoud Sadeghi, Santosh Kumar Tadakamadla

**Affiliations:** 1 Department of Orthodontics, School of Dentistry, Kermanshah University of Medical Sciences, Kermanshah 6713954658, Iran; 2 Interdisciplinary Department of Medicine, University of Bari “Aldo Moro”, 70124 Bari, Italy; 3 Department of Endodontics, School of Dentistry, Kermanshah University of Medical Sciences, Kermanshah 6713954658, Iran; 4 Department of Biology, Science and Research Branch, Islamic Azad University, Tehran 1477893855, Iran; 5 Dentistry and Oral Health, Department of Rural Clinical Sciences, La Trobe Rural Health School, La Trobe University, Bendigo, VIC 3550, Australia; 6 Violet Vines Marshman Centre for Rural Health Research, La Trobe Rural Health School, La Trobe University, Bendigo, VIC 3550, Australia

**Keywords:** dental caries, DEFB1 protein, MBL2 protein, polymorphism, meta-analysis

## Abstract

*Background and objective*: Some variants in *defensin beta 1* (*DEFB1*) and *mannose-binding lectin 2* (*MBL2*) genes can be associated with oral diseases. Herein, we designed a systematic review and meta-analysis to evaluate the association of *DEFB1* (*rs11362*, *rs1799946*, and *rs1800972*) and *MBL2* (*rs7096206* and *rs1800450*) polymorphisms with the susceptibility to dental caries (DC) in children. *Materials and methods*: A systematic literature search was conducted in the PubMed/Medline, Web of Science, Scopus, and Cochrane Library databases until 3 December 2022, without any restrictions. The odds ratio (OR), along with a 95% confidence interval (CI) of the effect sizes, are reported. Analyses including a subgroup analysis, a sensitivity analysis, and funnel plot analyses were conducted. *Results*: A total of 416 records were identified among the databases, and nine articles were entered into the meta-analysis. A significant relationship was found between the T allele of *DEFB1 rs11362* polymorphism and DC susceptibility, and the T allele was related to an elevated risk of DC in children (OR = 1.225; 95%CI: 1.022, 1.469; *p* = 0.028; I^2^ = 0%). No other polymorphisms were associated with DC. All articles were of moderate quality. Egger’s test in homozygous and dominant models demonstrated a significant publication bias for the association of *DEFB1 rs1799946* polymorphism with DC risk. *Conclusions*: The results demonstrated that the T allele of *DEFB1 rs11362* polymorphism had an elevated risk for DC in children. However, there were only few studies that evaluated this association.

## 1. Introduction

Oral health is important for a child’s overall health and development [1]. Dental caries (DC) or tooth decay involves damage to tooth enamel [2] and is a chronic disease that is widely prevalent [2,3]. DC is the most prevalent chronic disease among children [4,5,6], with 0.5 billion prevalent cases of caries in deciduous teeth among 0–14-year-old children [7].

Oral bacteria [8,9], dietary habits (sugar intake) [10], oral health behavior (e.g., toothbrushing and using fluoridated toothpaste) [11], feeding practices (e.g., breastfeeding practice and night bottle-feeding) [11,12], geographic area [13], and socioeconomic status (e.g., income, education, and social class) are factors that could serve as determinants of DC in children. In addition to these factors, genetic variations in the formation of enamel and immune response genes could be associated with a greater predisposition to DC [14]. Recent meta-analyses [15,16,17] reported a relationship between polymorphisms and DC risk.

The human beta-defensin 1 (DEFB1) is a 36-amino-acid antimicrobial peptide depending on the defensin family [18]. This peptide is encoded by the *DEFB1* gene and is the main molecule for protection from DC [19], and it has been detected in mucosal surface of airways, the gastrointestinal tract (esophagus, intestines, and stomach), urogenital tissue [20], salivary glands [21], and gingival and oral tissues [22]. The DEFB1 gene is associated with immune response, and researchers have shown that it acts as a host defense protein by influencing the non-specific immune system, as well as in adaptive immunity, thereby influencing DC progression [23,24].

Mannose-binding lectin (MBL) is a protein molecule inherent to the immune system, in which the activation of lectin (ubiquitous carbohydrate-binding protein) domains are found in relation to collagenous structures [25,26]. MBL insufficiency is one of the most common human immunodeficiencies and increases first from three single-point mutations in exon 1 of the *MBL2* gene [26]. Variations in this gene can be associated with DC [27].

Two meta-analyses [19,28] reported a relationship between polymorphisms and DC risk. One meta-analysis checked the relationship of *DEFB1* (*rs11362*, *rs1799946*, and *rs1800972*) and *MBL2* (*rs7096206, rs11003125,* and *rs1800450*) polymorphisms with DC risk [28] while the other just explored the role of *DEFB1 rs11362* polymorphism [19]. Both meta-analyses [19,28] analyzed the results, including individuals of all age groups. It is evident that age is a significant predictor of DC [29,30,31,32].

Although some researchers have reported selected polymorphisms of the *DEFB1* and *MBL2* genes to have an influence on the progression of DC, the results are still unconfirmed and inconsistent. We designed a systematic review and meta-analysis to evaluate the association of *DEFB1* (*rs11362*, *rs1799946*, and *rs1800972*) and *MBL2* (*rs7096206* and *rs1800450*) polymorphisms with the risk of DC in children with more studies for the first time in the English literature based on our knowledge.

## 2. Materials and Methods

To design the study, the PRISMA guidelines provided in the Supplementary file were followed [33]. The PECO (Population, Exposure, Comparator, and Outcome) question [34,35] was as follows: Is there an association between *DEFB1* and *MBL2* polymorphisms and susceptibility to DC? (P: Children with DC (CDC), E: DEFB1 and MBL2 polymorphisms, C: Children free of DC (CFC); O: DC).

### 2.1. Search Strategy and Study Selection

The Scopus, PubMed/Medline, Cochrane Library, and Web of Science databases were searched by one author (M.S.) to retrieve records published until December 3, 2022, without any restrictions (e.g., language). The keywords or search terms were (“beta defensin*” or “β-defensin*” or “beta-defensin 1” or “β-defensin-1” or “beta-defensin-1” or “defensin beta 1” or “DEFB1” or “human beta-defensin-1” or “HBD-1” or “mannose-binding lectin” or “MBL” or “mannose binding lectin 2” or “MBL2” or “mannose binding lectin-2” or “MBL-2” or “mannan-binding lectin” or “mannan-binding protein” or “MBP”) and (“tooth decay” or “dental caries” or “caries”). Moreover, the citations of the retrieved original articles/reviews/meta-analyses linked to the subject were searched to ensure that no study was missed. A second reviewer (G.H.) evaluated the titles/abstracts of the articles linked to the subject; afterwards, the full texts of the articles that met the inclusion criteria were downloaded and screened. Any study that was excluded was tagged with the reason for exclusion. In the event of a lack of agreement among the authors, a third reviewer (M.M.I) was involved.

### 2.2. Quality Assessment

The quality of studies was evaluated based on the modified Newcastle–Ottawa scale (NOS) [19] by two reviewers independently (G.H. and M.S.). The scores ranged from 0 to 10 points, with >7 points being considered as “high quality”, 4 to 7 points denoting “moderate quality”, and less than 4 points being “low quality”. Disagreement between the authors was resolved by a third reviewer (M.M.I.).

### 2.3. Eligibility Criteria

The inclusion criteria were as follows: (I) any type of articles including two independent groups (CDC and CFCs); (II) studies with any defined DMFT/dmft score for CDC and CFCs; (III) studies including polymorphisms of *DEFB1* or *MBL2* genes including minimum two studies for the analysis with any amount of the Hardy–Weinberg equilibrium (HWE); and (IV) CDC and CFCs had no chronic illnesses, genetic diseases, or other disorders. Irrelevant studies, meta-analyses, studies without a control group, studies with insufficient data for analysis, case reports, and conference papers were excluded.

### 2.4. Data Extraction

Two authors (S.B. and R.S.) independently extracted the data of the studies. Disagreement between the authors was resolved by a third author (P.C.)**.** The extracted data were the name of first author, publication year of the study, country of origin of the study, number of CDC and CFCs, ethnicity, age range of individuals, investigated dentition, DMFT/dmft score of the CDC and CFCs, type of reported polymorphism(s), the quality score of each study, and effect sizes (odds ratio (OR) and 95% confidence interval (CI)) for DC occurrence of each polymorphism according to five genetic models.

### 2.5. Statistical Analysis

To compute the effect sizes (ORs and 95% CIs) and the rest of the analyses, two authors (G.H. and M.S.) independently used comprehensive meta-analysis version 2.0 (CMA 2.0) software. Disagreement between the authors was resolved by a third author (M.M.I.). A *p*-value (two-sided) less than 0.05 was considered significant. The I^2^ statistic was used to estimate heterogeneity, with I^2^ > 50% (*P*_heterogeneity_ < 0.1) recommending a significant heterogeneity, and we used the fixed-effects model [36]. The publication bias across or among the studies was evaluated using Egger’s and Begg’s tests [37,38]; if *p*-value (two-sided) was less than 0.10 (two-sided) for one and both tests, a significant publication bias was considered to be present. With regard to the stability of the results, two sensitivity analyses, including ‘one-study-removed’ and ‘cumulative analyses’ were carried out. A subgroup analysis based on ethnicity, type of dentition, and sample size was carried out for *DEFB1 rs11362* polymorphism while such analysis wasn’t possible for *MBL2 polymorphisms* due to an insufficient number of studies.

## 3. Results

### 3.1. Study Selection

**Figure 1:**
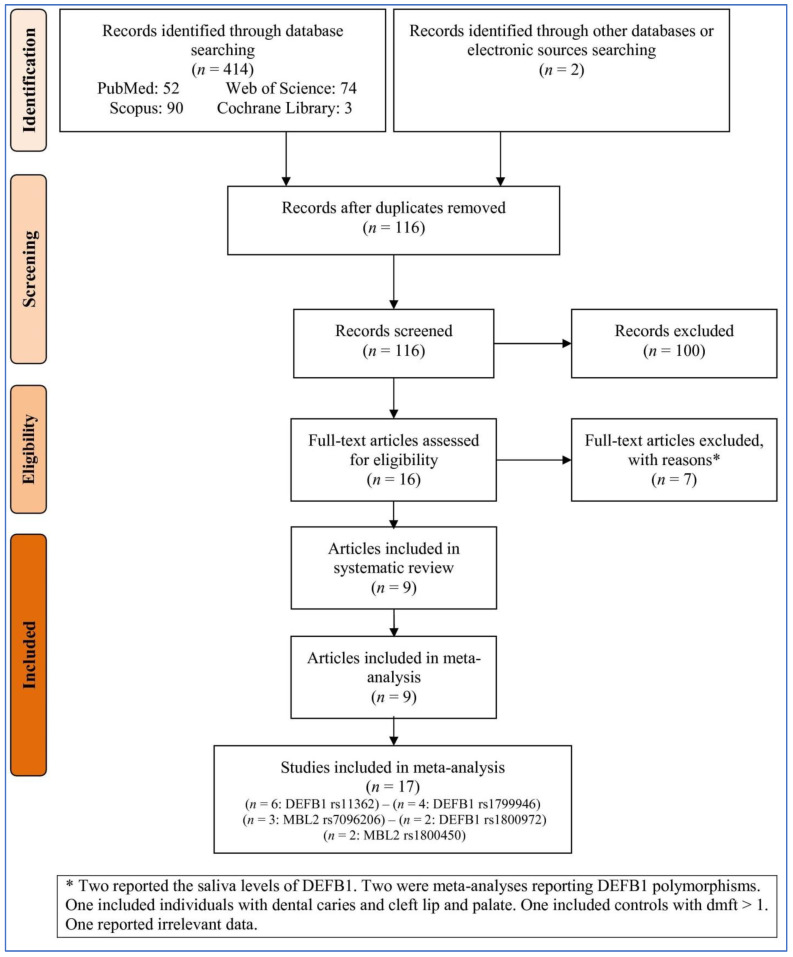
Flowchart of study selection. DEFB1: beta-defensin 1. MBL2: mannose-binding lectin 2.

By searching the databases and electronic resources, 416 records were identified. After removing duplicates and irrelevant records, 16 full-text articles were obtained and, after that, assessed. Among the evaluated full-text articles, seven were excluded for different reasons (Figure 1). Finally, nine articles [14,27,39,40,41,42,43,44,45] involving analyses of 17 studies (several articles included more than one polymorphism, and each polymorphism was considered one independent study) were involved in the meta-analysis.

### 3.2. Characteristics of the Studies

The articles were published from 2005 to 2020 (Table 1). Four articles [14,27,42,43] included Caucasians, three [40,44,45] Asians, and two [39,41] mixed ethnicities. Four articles [14,42,43,45] investigated deciduous dentitions, two [27,40] investigated permanent dentitions, two [41,44] investigated mixed dentitions, and one [39] investigated all three dentitions. Five and four articles evaluated the association of *MBL2* [27,40,43,44,45] and *DEFB1* [14,39,41,42] polymorphisms, respectively with DC risk.

### 3.3. Quality Assessment

Table 2 shows the quality evaluation of the articles by modified NOS. All articles [14,27,39,40,41,42,43,44,45] were of moderate quality.

### 3.4. Meta-Analysis

**Figure 2:**
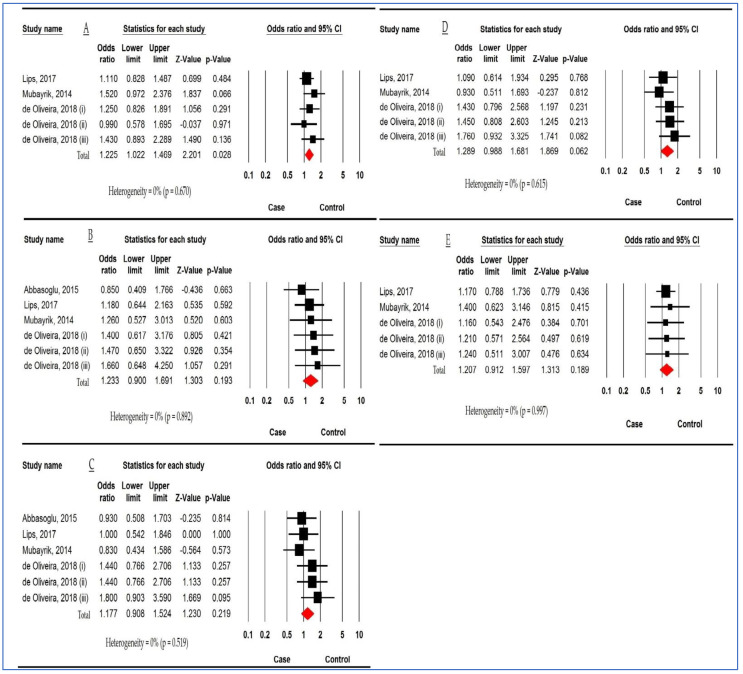
Forest plot showing the association between *defensin beta 1 (DEFB1) rs11362* polymorphism and dental caries risk. (**A**) Allelic model. (**B**) Homozygous model. (**C**) Heterozygous model. (**D**) Dominant model. (**E**) Recessive model. ((i) Deciduous dentition. (ii) Permanent dentition. (iii) Mixed dentition in the study of de Oliveira et al. [14,39,41,42]).

Figure 2 displays the relationship between *DEFB1 rs11362* polymorphism and DC risk based on six studies for homozygous and heterozygous models and five for other models. The pooled ORs were 1.225 (95%CI: 1.022, 1.469; *p* = 0.028; I^2^ = 0%), 1.233 (95%CI: 0.900, 1.691; *p* = 0.193; I^2^ = 0%), 1.177 (95%CI: 0.908, 1.524; *p* = 0.219; I^2^ = 0%), 1.289 (95%CI: 0.988, 1.681; *p* = 0.062; I^2^ = 0%), and 1.207 (95%CI: 0.912, 1.597; *p* = 0.189; I^2^ = 0%) in the allelic model (AM), the homozygous model (HoM), the heterozygous model (HeM), the dominant model (DM), and the recessive model (RM), respectively. There was only a significant relationship between the T allele of *DEFB1 rs11362* polymorphism and DC risk.

**Figure 3:**
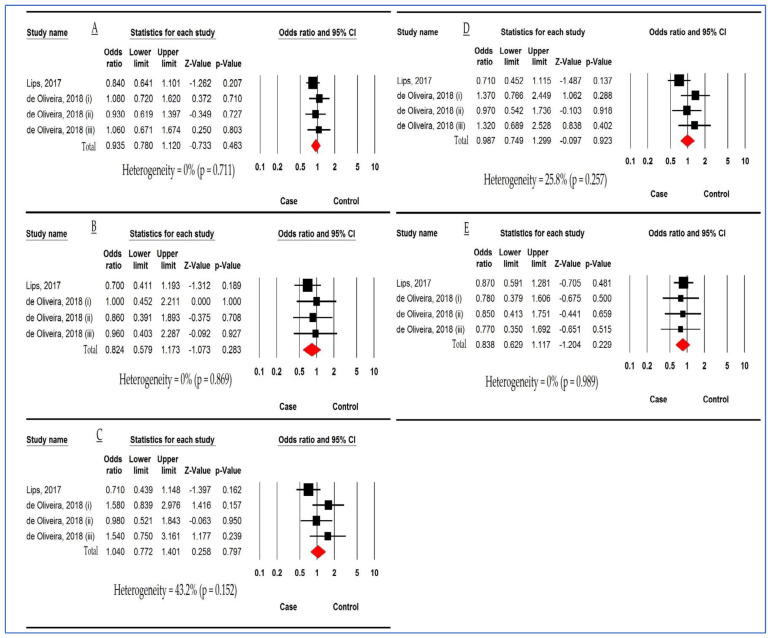
Forest plot analyses demonstrating the relationship between *defensin beta 1 (DEFB1) rs1799946* polymorphism and dental caries risk. (**A**) Allelic model. (**B**) Homozygous model. (**C**) Heterozygous model. (**D**) Dominant model. (**E**) Recessive model. ((i) Deciduous dentition. (ii) Permanent dentition. (iii) Mixed dentition in the study of de Oliveira et al. [39,41]).

Figure 3 reports the relationship between *DEFB1 rs1799946* polymorphism and DC risk based on four studies. The pooled ORs were 0.935 (95%CI: 0.780, 1.120; *p* = 0.463; I^2^ = 0%), 0.824 (95%CI: 0.579, 1.173; *p* = 0.283; I^2^ = 0%), 1.040 (95%CI: 0.772, 1.401; *p* = 0.797; I^2^ = 43.2%), 0.987 (95%CI: 0.749, 1.299; *p* = 0.923; I^2^ = 25.8%), and 0.838 (95%CI: 0.629, 1.117; *p* = 0.229; I^2^ = 0%) in AM, HoM, HeM, DM, and RM, respectively. There was no significant relationship between *DEFB1 rs1799946* polymorphism and the DC susceptibility in any of the five genetic models.

**Figure 4:**
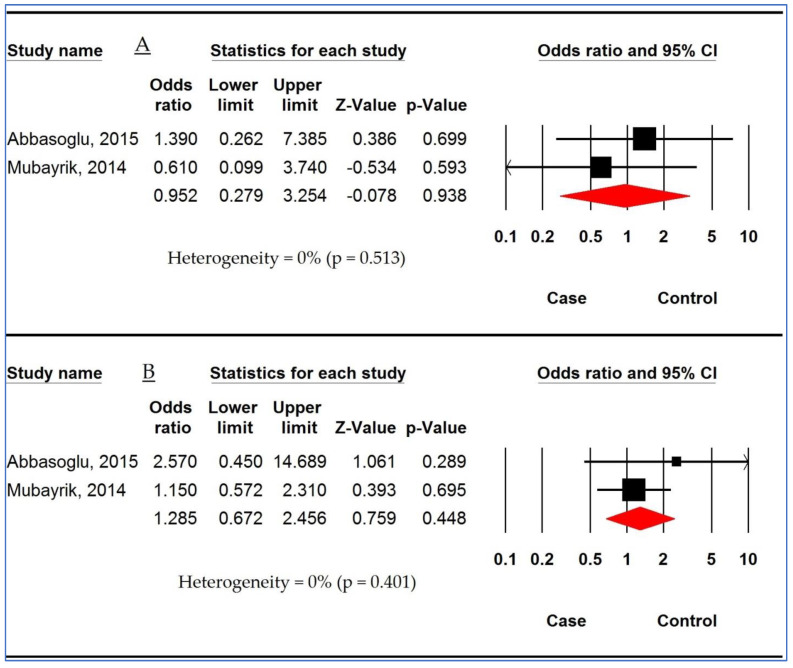
Forest plot for association between *defensin beta 1 (DEFB1) rs1800972* polymorphism and dental caries susceptibility. (**A**) Homozygous model. (**B**) Heterozygous model [14,42].

Figure 4 demonstrates the relationship between *DEFB1 rs1800972* polymorphism and DC susceptibility based on three studies in two models. The pooled ORs were 0.952 (95%CI: 0.279, 3.254; *p* = 0.938; I^2^ = 0%) and 1.285 (95%CI: 0.672, 2.456; *p* = 0.448; I^2^ = 0%) in HoM and HeM, respectively. There was no significant association between *DEFB1 rs1800972* polymorphism and susceptibility to DC.

**Figure 5:**
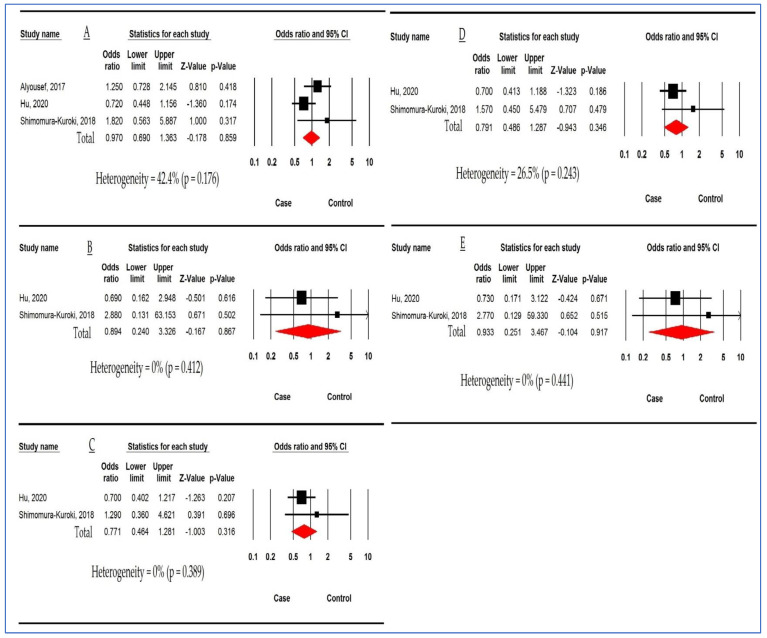
Forest plot for association between *mannose-binding lectin 2 (MBL2) rs7096206* polymorphism and dental caries risk. (**A**) Allelic model. (**B**) Homozygous model. (**C**) Heterozygous model. (**D**) Dominant model. (**E**) Recessive model [27,40,44].

Forest plot in Figure 5 demonstrates the association between *MBL2 rs7096206* polymorphism and DC susceptibility based on three studies in allelic models and two studies for other models. The pooled ORs were 0.970 (95%CI: 0.690, 1.363; *p* = 0.859; I^2^ = 42.4%), 0.894 (95%CI: 0.240, 3.326; *p* = 0.867; I^2^ = 0%), 0.771 (95%CI: 0.464, 1.281; *p* = 0.316; I^2^ = 0%), 0.791 (95%CI: 0.486, 1.287; *p* = 0.346; I^2^ = 26.5%), and 0.933 (95%CI: 0.251, 3.467; *p* = 0.917; I^2^ = 0%) in AM, HoM, HeM, DM, and RM, respectively. There was no significant association between *MBL2 rs7096206* polymorphism and susceptibility to DC.

**Figure 6:**
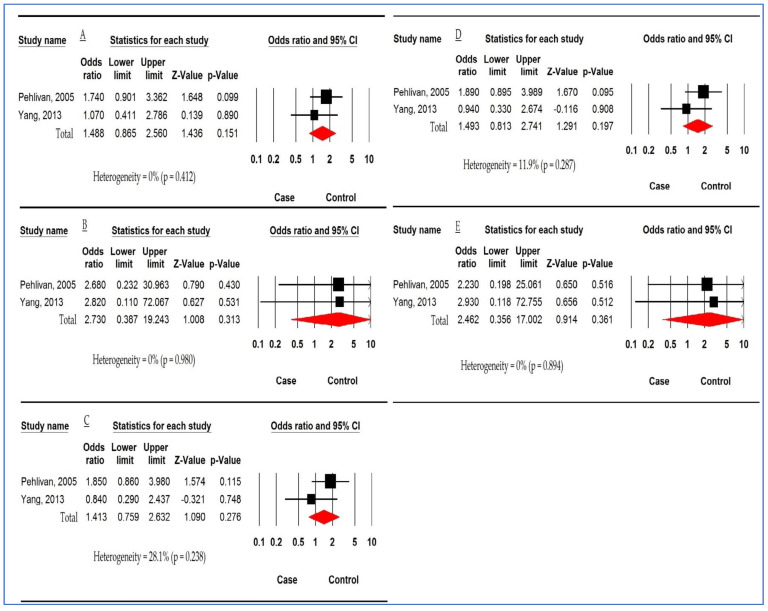
Forest plot for association between *mannose-binding lectin 2 (MBL2) rs1800450* polymorphism and dental caries susceptibility. (**A**) Allelic model. (**B**) Homozygous model. (**C**) Heterozygous model. (**D**) Dominant model. (**E**) Recessive model [43,45].

Forest plot in Figure 6 displays the association between *MBL2 rs1800450* polymorphism and DC susceptibility based on two studies. The pooled ORs were 1.488 (95%CI: 1.865, 2.560; *p* = 0.151; I^2^ = 0%), 2.730 (95%CI: 0.387, 19.243; *p* = 0.313; I^2^ = 0%), 1.413 (95%CI: 759, 2.632; *p* = 0.276; I^2^ = 0%), 1.493 (95%CI: 0.813, 2.741; *p* = 0.197; I^2^ = 0%), and 2.462 (95%CI: 0.356, 17.002; *p* = 0.361; I^2^ = 0%) in AM, HoM, HeM, DM, and RM, respectively. There was no significant association between *MBL2 rs1800450* polymorphism and susceptibility to DC.

### 3.5. Subgroup Analysis

Table 3 presents the subgroup analysis for the association between *DEFB1 rs11362* polymorphism and susceptibility to DC. Among ethnicity, type of dentition, and sample size in the five genetic models, the TT + CT genotype in mixed ethnicity and T allele in deciduous dentition had elevated risks of DC. Therefore, ethnicity and type of dentition were significant factors for the association between *DEFB1 rs11362* polymorphism and DC risk.

### 3.6. Sensitivity Analysis

The sensitivity analyses demonstrated stability of the results for all explorations where there were a minimum of three studies (results are not presented).

### 3.7. Publication Bias

The results of Egger’s and Begg’s tests were checked to evaluate the publication bias across the studies. The funnel plots are illustrated in the Supplementary file. The findings reported that just Egger’s test in homozygous and dominant models for association between *DEFB1 rs1799946* polymorphism and DC risk showed a significant publication bias.

## 4. Discussion

A meta-analysis [28] reported that among *DEFB1* (*rs11362*, *rs1799946*, and *rs1800972*) and *MBL2* (*rs7096206, rs11003125,* and *rs1800450*) polymorphisms, just *MBL2* rs11003125 had an association with the risk of DC, while another meta-analysis [19] found *DEFB1 rs11362* polymorphism to be associated with the risk of DC in permanent dentition, not deciduous or mixed dentitions. This systematic review evaluated the association of *DEFB1* (*rs11362*, *rs1799946*, and *rs1800972*) and *MBL2* (*rs7096206* and *rs1800450*) polymorphisms with DC risk; the findings suggest that *DEFB1 rs11362* polymorphism in T allele is related to an increased likelihood of DC occurrence. In addition, ethnicity and type of dentition were significant factors in the subgroup analysis checking the relationship of *DEFB1 rs11362* polymorphism with DC risk.

DC is a chronic disease that is usually affected by environmental and host agents and even genetic factors [2,46,47,48,49]. Therefore, early detection, early diagnosis, and early treatment are the main considerations for the prevention and treatment of DC [50,51].

There are many genetic agents that probably contribute to DC susceptibility and resistance, such as salivary agents, taste preference, tooth morphology, immune system, enamel structure and composition, organic and inorganic substances, and behavior [16,17,46,52,53,54]. In addition, the likelihood of DC occurrence is high in the first months after the tooth eruption but is much lower in adulthood and later stages of life, and at different ages, the DC intensity may be different [32].

Two studies [27,40] reported that the differences between the results of studies reporting the relationship between polymorphisms and susceptibility to DC can be a result of the variation in the sample sizes, experimental methods, and ethnicities. Another study [55] that included 53 genes reported as being involved in DC susceptibility showed that cytokine network relevant genes, the transforming growth factor-beta family, and the matrix metalloproteinases family had important roles in tooth development and carious lesions. It is believed that the flow of saliva, pH, and chemical composition of saliva are among other important factors in the occurrence and progress of DC [56,57,58]. The present meta-analysis reported that ethnicity and type of dentition were important factors for the relationship of *DEFB1 rs11362* polymorphism with susceptibility to DC in children. Other reports showed that Africans had 32% and Mixed ethnicities had 69% more DC experience than Whites [59] and that DC prevalence was 30.4%, 39.0%, and 51.7% for White, Black, and Hispanic students, respectively [60]. For *DEFB1* (*rs1799946* and *rs1800972*) and *MBL2* (*rs7096206* and *rs1800450*) polymorphisms, we could not perform a subgroup analysis due to the limited number of reported studies. Therefore, a large number of studies are needed to prove the role of ethnicity in the prevalence of tooth decay. This can be due to the difference in ethnical factors (geographical conditions, bone structure, nutrition, etc.) that have affected dental genetics over time. To find these relationships, further studies and further emphasis on ethnicity and the risk of DC in the future can be discussed in further possible mechanisms. With regard to the type of dentition, the etiology of dental anomalies is partly environmental and partly genetic [61] and the DC phenotypes in the deciduous dentition were highly heritable [62]. Therefore, the role of type of dentition can be affected by genetics, but more studies are needed to find the possible mechanisms between type of dentition and risk of DC.

DEFB1 as an oral antimicrobial peptide gives the first line of defense against an extensive range of pathogens [63,64]. The high variability of defensin levels in oral tissues can be attributed to genetic changes in the host [65,66]. The present meta-analysis reported that *DEFB1 rs1799946* polymorphism was related to the elevated risk of DC in children, and therefore, this can cause a reduction in DEFB1 and then oral infections. DC is caused by bacteria that destroy the enamel and dentin [67,68,69]. Therefore, the role of *DEFB1* polymorphisms could be considered in future studies for reaching better and more accurate results.

The *MBL2* plays an important role in the innate immune system and few polymorphisms in this gene can be responsible for increased susceptibility to some infectious diseases [70,71,72]; therefore, MBL2 insufficiency is related to bacterial infection [73]. The present meta-analysis could not find any association between *MBL2* (*rs7096206* and *rs1800450*) polymorphisms, perhaps due to a limited number of included studies. Therefore, more studies are needed to support or reject the present meta-analysis results.

The role of oral peptides as therapeutic agents and for clinical assessment of an individual’s susceptibility to DC can be promising in the future [74,75,76]. Oral antimicrobial peptides give the first line of defense against an extensive range of pathogens [75,77]. Their expression in saliva and all over the oral cavity denotes their role in preserving the tooth structure from DC, as well as preserving the oral mucosa, in spite of the fact that the amount of antimicrobial peptides expressed in saliva varies among people [66,76,78,79]. Therefore, paying attention to the metabolism pathways of peptides, their genetic mutations, the values of these peptides in blood and saliva, and their expression can greatly help future research in finding factors associated with susceptibility to DC.

This meta-analysis has three limitations: (1) There were a limited number of published studies, therefore, an inability to conduct subgroup analyses for most polymorphisms; therefore, more studies with more cases are needed to confirm the association of these polymorphisms with DC risk. (2) There was possibly a publication bias for some analyses, which could also be due to fewer studies being included in the analyses. (3) None of the studies were of high quality. In contrast, the stability of the results and low/lack of heterogeneity across the studies were the strengths of the meta-analysis.

## 5. Conclusions

The findings suggest that the T allele of *DEFB1 rs11362* polymorphism is associated with an increased likelihood of DC. Therefore, this polymorphism could have a significant role in the pathogenesis of DC. The limited number of studies and the moderate quality of the included studies demonstrate that well-designed studies with more cases are needed to confirm or reject the results.

## Figures and Tables

**Table 1:** Features of articles included in the analysis.

First Author, Publication Year	Country	Ethnicity	No. of Cases	No. of Controls	Age Range, Years	Investigated Dentition	Caries Index (Control; Case)	Polymorphisms
Pehlivan, 2005 [43]	Turkey	Caucasian	42	40	< 18	Deciduous	dmft (0; NR)	*MBL2 rs1800450*
Mubayrik, 2014 [42]	Turkey	Caucasian	87	74	2 to 6	Deciduous	dmft (0; ≥1)	*DEFB1 rs11362* *DEFB1 rs1800972*
Yang, 2013 [45]	China	Asian	70	70	1 to 5	Deciduous	dmft (0; ≥1)	*MBL2 rs1800450*
Abbasoğlu, 2015 [14]	Turkey	Caucasian	136	123	2 to 5	Deciduous	dmft (0; ≥1)	*DEFB1 rs11362* *DEFB1 rs1800972*
Alyousef, 2017 [27]	Saudi Arabia	Caucasian	204	200	5 to 13	Permanent	DMFT (0; NR)	*MBL2 rs7096206*
Lips, 2017 [41]	Brazil	Mixed	87	81	2 to 12	Mixed	DMFT/dmft (0; ≥4)	*DEFB1 rs11362* *DEFB1 rs1799946*
de Oliveira, 2018 [39]	Brazil	Mixed	117	78	10 to 12	Deciduous	DMFT/dmft (0; ≥1)	*DEFB1 rs11362*
			118	78		Permanent		
			265	49		Mixed		
			117	78	6 to 12	Deciduous		*DEFB1 rs1799946*
			118	78		Permanent		
			265	49		Mixed		
Shimomura-Kuroki, 2018 [44]	Japan	Asian	53	28	3 to 11	Mixed	DMFT/dmft (0; ≥1)	*MBL2 rs7096206*
Hu, 2020 [40]	China	Asian	198	162	12 to 15	Permanent	DMFT (0; ≥1)	*MBL2 rs7096206*

NR: not reported. DEFB1: beta-defensin 1. MBL2: mannose-binding lectin 2. dmft: decayed, missing and filled primary teeth. DMFT: decayed, missing, and filled permanent teeth.

**Table 2:** Quality evaluation of the articles by modified Newcastle–Ottawa scale.

First Author, Publication Year	Representativeness of Cases	Source of Controls	Hardy–Weinberg Equilibrium in Controls	Genotyping Examination	Association Assessment	Total Score
Pehlivan, 2005 [43]	*	* *	**	-	* *	7
Mubayrik, 2014 [42]	*	*	* *	-	* *	6
Yang, 2013 [45]	*	*	*	-	* *	5
Abbasoğlu, 2015 [14]	*	* *	*	-	* *	6
Alyousef, 2017 [27]	*	*	**	-	* *	6
Lips, 2017 [41]	*	* *	*	-	* *	6
de Oliveira, 2018 [39]	*	* *	*	-	* *	6
Shimomura-Kuroki, 2018 [44]	*	*	**	-	* *	6
Hu, 2020 [40]	*	*	**	-	* *	6

Each asterisk denotes 1 score.

**Table 3:** Subgroup analysis for *beta-defensin 1 (DEFB1) rs11362* polymorphism.

Variable	Model, N	OR	95%CI	*p*-Value	I^2^, %
Ethnicity					
Caucasian	Allelic (1)	1.520	0.972, 2.376	0.066	-
	Homozygous (2)	1.000	0.571, 1.751	1.000	0
	Heterozygous (2)	0.882	0.567, 1.372	0.578	0
	Dominant (1)	0.930	0.511, 1.693	0.812	-
	Recessive (1)	1.400	0.623, 3.146	0.415	-
Mixed	Allelic (4)	1.157	0.964, 1.432	0.111	0
	Homozygous (4)	1.360	0.928, 1.993	0.115	0
	Heterozygous (4)	1.368	0.994, 1.883	0.055	0
	Dominant (4)	1.396	1.038, 1.878	**0.028**	0
	Recessive (4)	1.182	0.877, 1.594	0.272	0
Dentition					
Deciduous	Allelic (2)	1.368	1.010, 1.854	**0.043**	0
	Homozygous (3)	1.113	0.701, 1.768	0.649	0
	Heterozygous (3)	1.036	0.722, 1.489	0.846	0
	Dominant (2)	1.159	0.762, 1.762	0.490	1.3
	Recessive (2)	1.267	0.728, 2.203	0.403	0
Permanent	Allelic (1)	0.990	0.578, 1.695	0.971	-
	Homozygous (1)	1.470	0.650, 3.322	0.354	-
	Heterozygous (1)	1.440	0.766, 2.706	0.257	-
	Dominant (1)	1.450	0.808, 2.603	0.213	-
	Recessive (1)	1.210	0.571, 2.564	0.619	-
Mixed	Allelic (2)	1.191	0.929, 1.527	0.167	0
	Homozygous (2)	1.304	0.784, 2.170	0.307	0
	Heterozygous (2)	1.296	0.819, 2.049	0.268	35.8
	Dominant (2)	1.351	0.882, 2.068	0.166	16.8
	Recessive (2)	1.181	0.824, 1.694	0.365	0
Sample size					
≥200	Allelic (1)	1.430	0.893, 2.289	0.136	-
	Homozygous (2)	1.094	0.614, 1.948	0.760	17.6
	Heterozygous (2)	1.239	0.786, 1.953	0.356	49.7
	Dominant (1)	1.760	0.932, 3.325	0.082	-
	Recessive (1)	1.240	0.511, 3.007	0.634	-
<200	Allelic (4)	1.193	0.980, 1.451	0.078	0
	Homozygous (4)	1.298	0.890, 1.893	0.175	0
	Heterozygous (4)	1.148	0.837, 1.573	0.392	0
	Dominant (4)	1.206	0.900, 1.617	0.209	0
	Recessive (4)	1.203	0.895, 1.617	0.220	0

OR: odds ratio. CI: confidence interval. N: number of studies. Bolded numbers mean statistically significant.

## Data Availability

No new data were created or analyzed in this study. Data sharing is not applicable to this article.

## Supplementary Materials

The following are available online at https://www.mdpi.com/article/10.3390/children10020232/s1.
